# Coronary Artery Disease in Women: An Unsolved Dilemma

**DOI:** 10.14740/jocmr1725w

**Published:** 2014-02-06

**Authors:** Fahad Aziz

**Affiliations:** Penn State Hershey Medical Center, Hershey, PA 17033, USA. Email: fahadaziz.md@gmail.com

**Keywords:** Coronary artery disease, Women, Management

## Abstract

Cardiovascular disease (CVD) is the leading cause of death in women, as well as an important cause of disability, although many women and their physicians underestimate the risk. The pathogenesis, presentation and diagnosis of CVDs are different in women than men, which make the women prone to under-treatment for these diseases. More gender-based research regarding the management of coronary artery disease (CAD) in women needs to be done. Exercise, hypertension treatment, smoking cessation and aspirin therapy are effective measures for the primary prevention of CAD in women. The roles of hormone replacement therapy in primary prevention are not well established. Hormone replacement therapy has not been effective in lowering the risk of recurrent myocardial infarction. Cardiologists and family physicians should emphasize the use of proven treatments, with particular attention given to underserved populations.

## Introduction

Cardiovascular diseases (CVDs) are the most frequent causes of death in both males and females throughout the world. The importance and severity of coronary artery disease (CAD) in women are always underestimated. In the USA, more than 250,000 women are dying from CAD every year. In the premenopausal time, women have extra protection from CAD, as evident by a 10- to 20-year delay in the onset of CAD among women when compared to men [1, 2]. Recent data showed that mortality and morbidity of the women with regard to CAD are rising significantly since 1980 [3]. This situation is even worse in the developing countries. Women in these developing countries are at increased risk of CVD when compared to their American counterpart. The data collected from India and china also support the observation of early onset of ischemic heart disease in Asian women [4-9].

CAD has been prevalent among women but it is only recently that is being cautiously scrutinized as described in the medical literature [10]. The misconception that CAD and arteriosclerotic disease are common among the men and women are naturally protected against them is vastly prevalent.

Even after the presentation of these facts by many studies, the attitude of cardiologists and physician has not changed towards the tackling of women heart disease. Most of the research-based evidence regarding treatment of CAD has been gathered by experimenting the male population that makes it questionable, whether we should implement the same guidelines on the women [11].

Many of the sex-based researches have shown that women have different pathogenesis, clinical presentation and complication related to CAD as compared to the males.

Vaccarino et al have also provided new evidence, based on analysis of large databases, that there are differences between men and women in the natural history of CAD that are not related to age [12]. More research-based evidence is needed to familiarize the physicians with these differences to set up the new guidelines for the women-based management of the heart diseases.

## Gender Differences in Pathophysiology of CAD

The pathophysiology of atherosclerosis is, clearly, different in women when compared to the men. The women have a higher risk of blood coagulability making them at high risk for the blood clot formation.

In large number of women endothelial dysfunction, small vessel size and diffuse atherosclerosis have been identified as causes of ischemia without evidence of blockade in the coronary arteries [13].

Further, atherosclerotic plaque in women is less fibrotic and contains more lipid filled foam cells, implying greater potential for reversibility but also potentially greater vulnerability for plaque rupture and thrombosis [11].

Women have similar magnitude of atherosclerosis, but it looks and functions differently possibly due to estrogen or genetic related reasons [11]. These different mechanisms in women result in different clinical presentations of CAD in women.

## Gender Differences in Coronary Risk Factors

Manson et al showed women have more multiple risk factors (42% vs. 20%), and few women are without traditional risk factors (3.5% vs. 14.3%) as compared to men [14].

Average elevated total cholesterol and LDL are important risk factors in men but are only weakly associated with CAD in women. Low HDL and high triglycerides are better causes of coronary risk in women than high concentration of LDL [14].

Smoking is more commonly associated with CAD in men, but if women smoke and use oral contraceptives, smoking will significantly increase the risk of CAD and deep venous thrombosis.

Depression has been found to be an important cause for complications after myocardial infarction (MI) and is seen more prevalent in female patients.

Some studies have increasingly shown that the traditional risk factors underestimate CHD risk in women. Both CRP and LDL-C show strong linear relationship with cardiac events, with CRP being the stronger predictor. These data suggest that CRP shows promise when added to traditional risk factors for prediction of long-term risk [15].

## Gender Differences in Clinical Manifestations of CAD

Lori et al showed women presentation of CAD is different from the men. Women usually present more with unstable angina (37%vs.27%) than NSTEMI, STEMI and sudden death (62% vs.42%), which are more common presentations in men. Acute coronary syndrome (ACS), a manifestation of CAD is more often clinically silent or misdiagnosed in women than in men (35% vs. 28%) because of variation in the presenting symptoms. EKG is less likely to show significant changes in women (37% vs. 27%) [4]. Cardiac biomarkers like BNP and CRP are better predictors of ischemic events in women as opposed to the routinely checked CKMB and troponins, which do not rise to high levels.

## Gender Differences in Provision of Care During Hospitalization

There are no significant differences among the two sexes as far as response to treatment benefits is concerned.

It has been seen that women experience more complications from diagnostic angiography and have poorer result after angioplasty. Fewer women get the bypass surgery and when they do undergo the surgery, they are less likely to receive LIMA grafts. Complications after surgery like heart failure, peri-operative MI, hemorrhage, neurologic and vascular issues are more frequent in women, which may be due to a woman’s older age, smaller body size, greater severity of angina, small and more fragile vessels, and greater burden of associated coronary risk factors. As a conclusion, there are different risks and benefits with commonly used therapies given to women for CAD.

## Morbidity and Mortality

Christine et al showed that the women have higher rate of severe complications during hospitalization (25% vs.11%) [11]. Sixty-three percent of women are seen to have sudden death while 42% women (vs.24% men) die within one year of a heart attack [11].

## Evaluation of Cardiovascular Risk

Medical history, family history, pregnancy complication history and symptoms of CVD should be evaluated. Along with that there should be depression screening in women with CVD, physical examination including BP, body mass index, waist size, laboratory test for fasting lipoproteins and glucose, and Framingham risk assessment/score if no CVD or diabetes.

## High Risk

High-risk women for CVD are those who have more than one of the following:

1. An established coronary artery disease
2. Carotid artery stenosis
3. Peripheral arterial disease
4. Abdominal aortic aneurysm
5. Chronic renal disease (especially ESRD).

## Intermediate Risk

1. Women with metabolic syndrome
2. Age above 60 years
3. A first degree relative with premature CVD
4. Women with subclinical cardiovascular disease (e.g. elevated coronary calcium score).

## Lifestyle Modifications

It is important for all women regardless of risk status to adopt a healthy lifestyle as early as possible.

### Smoking

Smoking is a major risk factor for the development of CVD in women. More than 60% of MIs in women younger than 50 years are attributable to smoking, as are 21% of all deaths from CAD. All women benefit from quitting smoking. Investigators in the Nurses’ Health Study found that the risk of CAD decreases by one-third, 2 years after smoking cessation in women [16].

In the United States, fewer women than men currently smoke. Compared with male smokers, women more often smoke to relieve stress, anger, boredom or depression. Women are more likely than men to smoke as a strategy for weight loss, and they more often give weight gain as a major reason for relapsing after quitting [17].

Compared with men, women find it harder to quit smoking initially, with and without treatment, and are also more likely to relapse. Some evidence shows that nicotine replacement appears to be more effective in men when compared to the women [18].

Because of gender-based differences in reasons for smoking, reasons for quitting and responses to pharmacologic agents, research on gender-specific smoking cessation strategies is needed. For example, it might be helpful for women to quit smoking early in their menstrual cycle (when withdrawal symptoms are not overlapping with premenstrual symptoms), to use bupropion to minimize weight gain and to address psychosocial stressors. Consideration of factors unique to women may help physicians be more successful in treating women smokers.

### Heart healthy diet

A healthy weight through a balanced diet that includes fruit and vegetables, whole grains, low fat or non-fat dairy, legumes, low fat protein and fish, is an irreplaceable step towards diminishing CVD. Saturated fats should be < 10% of calories and < 300 mg cholesterol. Low fat diet tends to raise triglyceride and lower HDL. The new NCEP guidelines favor a 25% to 35% fat diet, with only saturated fat restriction [11]. Limit trans fatty acid intake (sources are baked goods and fried foods made with partially hydrogenated vegetable oil). It is advisable to substitute animal fats (butter, and so on) with soft margarines and monounsaturated oils (olive or canola), or polyunsaturated oils (corn or sunflower), and to limit fried food, fatty meats and salt intake. Ideal weight should be between 18.5 and 24.9 BMI and waist circumference < 35 inches/or 38cm. Weight loss goals should be achieved by reducing 10% of body weight over 6 months or 1-2 pounds weight loss/week and reduce calories by 500-1,000 per day and at the same time treat all the components of metabolic syndrome, if present in that patient.

### Cardiac rehabilitation

High-risk women with a recent acute CV event, coronary intervention, new onset or chronic angina should participate in a comprehensive risk reduction program, such as cardiac rehabilitation, or a physician-guided home or community-based program along with associated risk factor control according to guidelines.

### Psychosocial factors

Women with CVD should be evaluated for depression and should be referred and treated when indicated.

## Preventive Drug Interventions for Women With CVD

### Aspirin and other cardio-protective drugs

Investigators in the Nurses’ Health Studyobserved a decreased risk of first MI and death in women who took 1-6 aspirins per week [19].

In the hypertension optimal treatment (HOT) study, men (53%) and women (47%) were randomized to receive 75 mg of aspirin per day or placebo, in addition to hypertension treatment [20]. The HOT study showed an association between aspirin use and reductions in major cardiovascular events and MIs.

The use of aspirin therapy for primary prevention of CVD appears to be justified in women with hypertension and other risk factors. However, further studies are needed to determine the optimal aspirin dose and whether other groups of women are likely to benefit from this treatment.

Aspirin therapy is clearly beneficial in women with chronic stable angina, unstable angina and evolving MI, and for the prevention of recurrent infarction.

Women, especially if older, African American or Hispanic, are less likely than white men to receive aspirin, beta-blockers, angiotensin-converting enzyme inhibitors and lipid-lowering drugs after MI, despite evidence of benefit [21].

These patients are also less likely to be referred for revascularization proceduresand cardiac rehabilitation [22].

## Intervention That are Not Useful/Effective and May be Harmful for the Prevention of Heart Disease

### Hormonal replacement therapy

Investigators in many observational studies found decreases in CAD outcomes among women receiving hormone replacement therapy. However, observational studies suffer from “healthy user” bias: women who are receiving hormone replacement therapy are more likely to be of higher socioeconomic status, to be healthier, to seek medical care and to exercise. These studies were not designed to detect the possible adverse effects of hormone therapy. In the prospective estrogen-progesterone interventions (PEPI) trial, estrogen replacement improved lipid profiles [23].

However, the PEPI trial was not designed to evaluate the effects of this treatment on the incidence and mortality of CAD.

The heart and estrogen/progestin replacement study (HERS) was a randomized, controlled trial of combined continuous hormone replacement therapy as secondary prevention in women with known CAD. In the HERS hormone replacement group, the rate of coronary events increased in the first two years and decreased in the third and fourth years, with no net benefit over the study period [24].

The possible increased risk of breast cancer in women who receive hormone replacement therapy remains a concern [25].

Estrogen replacement has proven benefits for reducing peri-menopausal symptoms and preventing osteoporosis. At present, however, there is insufficient evidence to recommend hormone replacement therapy for the prevention of coronary artery CAD.

### Antioxidant supplements

No cardiovascular benefits in randomized trials of primary and secondary prevention.

### Folic acid supplementations

Folic acid supplementations may be considered in high-risk women if a higher than normal level of homocysteine has been detected.

### OMEGA-3 fatty acids

OMEGA-3 fatty acids can be added as an adjunct to diet. Omega-3 fatty acid in the form of fish or capsules, for the primary and secondary prevention of hypercholesterolemia and/ hypertriglycerdeamia supplements may be considered in high-risk women.

## Conclusion

There is a tremendous need for action regarding knowledge and public understanding of CAD so that this issue may be effectively dealt with. At a time when health information is easily accessible and widely spread, many women are still not aware that CAD is, infect, one of the most important quencher of women’s lives. Adoption and application of new knowledge regarding sex differences will hopefully lead to improved results in women medicine.

Some, yet important, positive steps have been seen in the face of published articles, in the press (read by common man) as well as in medical journals, about the importance of cardiovascular health in women.
